# Dye removal membrane from electrospun nanofibers of blended polybutylenesuccinate and sulphonated expanded polystyrene waste

**DOI:** 10.1038/s41598-023-42424-3

**Published:** 2023-09-18

**Authors:** Norhan Nady, Mona H. Abdel Rehim, Abdelrahman A. Badawy

**Affiliations:** 1 Polymeric Material Research Department, City of Scientific Research and Technological Applications (SRTA-City), New Borg El-Arab City, 21934 Alexandria Egypt; 2 Packaging Materials Department, National Research Center, Institute of Chemical Industries Research, 33 El Behooth St., Dokki Giza, Egypt; 3 Physical Chemistry Department, National Research Centre, Advanced Materials Technology and Mineral Resources Research Institute, Giza, 12622 Egypt

**Keywords:** Chemistry, Polymer chemistry

## Abstract

Polystyrene (PS) is a thermoplastic polymer used in food packaging and the manufacture of trays and cups, among other applications. In this work, the preparation of a membrane by electrospinning blended sulphonated expanded PS waste and polybutylenesuccinate (PBS) is described. The fiber quality is controlled by selecting the right polymers’ ratios and solvents. Investigation of the structure of the produced membranes by Fourier transform infrared spectroscopy-attenuated total reflectance confirmed the successful sulphonation of expanded PS and the appearance of characteristic (PBS) bands in the prepared blends. Morphology study of the electrospun membranes using a scanning electron microscope revealed that the quality of the fibers is improved significantly by increasing the amount of PBS in the blend solution. Moreover, continuous and more homogenous fibers are produced by increasing the ratio of PBS to 2%. The efficiency of the prepared membranes in dye removal was tested using methylene blue. The effects of different parameters such as, pH, contact time, temperature, and dye concentration have been studied. Also, kinetic and adsorption isotherm models as well as the durability of the prepared membranes were investigated. The membrane prepared from PSS/1% PBS demonstrated the highest dye uptake (846 mol) with good regeneration efficiency. The adsorption process was found to be endothermic and fits the Freundlich isotherm and pseudo-second-order kinetic model. The values of activation energy for the adsorption process are 36.98, 30.70, and 43.40 kJ/mol over PSS, PSS/1% PBS and PSS/2% PBS, respectively.

## Introduction

Polystyrene (PS) is a linear thermoplastic resin widely used in our daily lives due to its low production cost, lightweight and being odorless, and colorless. It is largely commercialized as a hard and thermally stable material. Polystyrene is used in a wide variety of industries, including food packaging, medical equipment, automobiles, and electric and electronic gadgets^1^. The chemical structure of PS includes phenyl rings and C–C single bonds, making the polymer highly stable and resistant to UV radiation, and consequently, its waste is nondegradable. The majority of used PS is disposed of as a waste material, while a small amount enters the recycling process after modification. Nevertheless, recycling PS is still immature due to its unique properties and high stability. Modification of waste PS by the introduction of sulfonic groups to PS chains enables changing it into a polymer of different chemical, physical, and mechanical properties. The sulfonated polymer reveals different solubility and hydrophilicity characteristics^2^. Moreover, polystyrene sulphonate (PSS) as a polyelectrolyte, has found a wide range of applications^3^. The possibility of controlling its structure led to the obtaining of PSS, with versatile properties that have been explored in water treatment, and dye removal^4–8^.

Electrospinning is a widely used technique to fabricate fibers of varying diameters at the micro or nanoscale from polymer solutions^9^. It was reported that electrospinning polyelectrolytes is difficult due to repulsive forces between ionic groups present on the polymeric chains^10^. However, the synthesis of many polyelectrolytes by electrospinning has been described such as sulfonated poly(ether ether ketone)^11^, poly(acrylic acid)^12^, and sulfonated poly(arylene ether sulfone)^13^, etc.^14^. Electrospun PSS has been proposed for many applications, among them sensors^15^ and proton exchange membranes for fuel cells^16^. Cross-linked PSS mats were fabricated by electrospinning PS and then treated with chlorosulphonic acid^17^.

Dyes are coloring compounds used in a variety of industries, including paper, paint, leather, and textiles^18^. Due to their great chemical stability and minimal biodegradation, these dyes have detrimental effects when disposed of in the environment^19^. Moreover, they have a dangerous effect on human health. The bad effects of these dyes are not limited to humans but also occur even at small concentrations in animals^19^. Disposal of these dyes has been raised as an important task, particularly for facing the crisis of water shortages all over the world.

Many techniques have been used for dye removal, such as precipitation and filtration, photocatalysis, reverse osmosis, evaporation, electrochemical oxidation, solvent extraction, ozonation, flotation, and ion exchange^20–27^. These techniques suffer from many drawbacks during application^28–30^. Nowadays, the utilization of the adsorption technique is accelerated since it is a cheap, great-capacity process with good regeneration aptitude and simplicity^31^. Therefore, researchers still try to find suitable adsorbents to remove contaminants from wastewater^32–45^. Utilizing cobalt-ferrite magnetic nanocomposites based on sulfonated waste polystyrene for effective Calcon dye degradation, was reported^45^. The method enables utilizing discarded polystyrene to reduce water contamination, which lessens environmental pollution. Wahyuni et al. described removal of methylene blue dye using magnetized sulfonated polystyrene^40^. The results demonstrated that the adsorbent containing 50% Fe_3_O_4_ has good separability and adsorption efficiency. Moreover, the Langmuir isotherm model, which has an adsorption capacity of 46.56 mg/g, is a good fit for the adsorption kinetics.

In this context, this work describes the use of polystyrene waste for the preparation of an electrospun membrane for the dye removal from contaminated water. Firstly; sulfonation of waste polystyrene was carried out, then it was blended with different ratios of polybutylene succinate to obtain the membrane dope. The chemical structure of the electrospun membranes was studied by Fourier transform infrared spectroscopy attenuated total reflectance, while their surface morphologies have been investigated using scanning electron microscope imaging. Thermogravimetric analysis was used to study the thermal stability of the membranes. The efficiency of the prepared membranes for Methylene Blue removal was tested. The effects of many parameters such as, pH contact time, temperature, and dye concentration were investigated. Moreover, kinetic and adsorption isotherm models, as well as the durability of the prepared membranes, were thoroughly studied.

## Experimental section

### Materials

**Figure 1:**
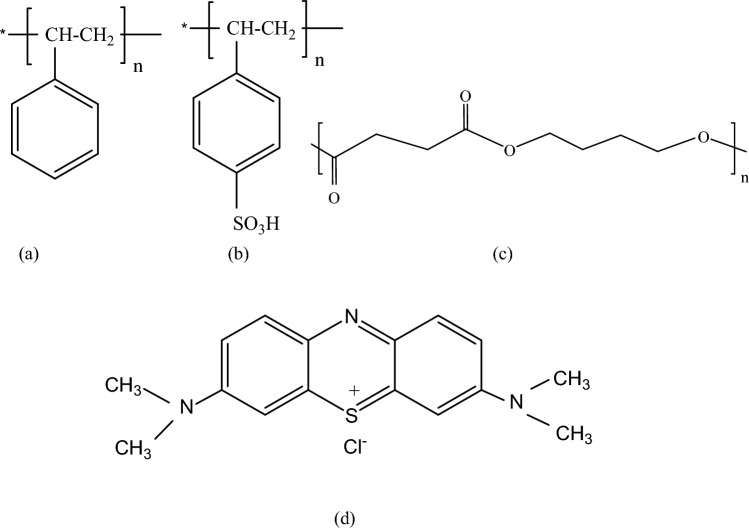
Chemical structure of (**a**) polystyrene (PS), (**b**) polystyrene sulfonate (PSS), (**c**) Polybutylene succinate (PBS), and (**d**) Methylene blue dye.

Expanded (PS), a commercial recycled polymer, Polybutylene succinate (PBS), 50% bio-sourced, (reference PBE 003 BB) was supplied by Natureplast© (Ifs, France). Sulfuric acid was a product of ADWIC Co (Egypt). Toluene and Chloroform (> 99%) were obtained from Fisher (United Kingdom), 1,4-Dioxan (> 99.5%) was purchased from Elnasr pharmaceutical chemicals co. (Egypt). Lithium chloride was obtained from Sigma-Aldrich (> 99%). Methylene blue (Fig. 1d) was purchased from Sd Fine-Chem Limited (India).

### Methods

#### Sulfonation of polystyrene

The crushed polystyrene foam (20 g) is dissolved in 200 mL of toluene. 2 mL of fuming sulfuric acid is added to the polystyrene solution, and the reaction proceeds at 60 °C for 2 h with continuous stirring. The sulfonation reaction was stopped by pouring the solution onto a large amount of distilled water (500 mL). A white, milky solution was formed, and the modified polymer was separated using a rotary evaporator. The excess sulfuric acid was removed by washing the polymer several times until the neutral pH of the washing wastewater was reached, and finally the polymer was dried in an oven at 80 °C.

#### Membrane dope preparation

The blend membrane dope was prepared using (6.5/1) and (5.5/2) wt% of PSS and PBS polymers, respectively, dissolved in dioxane: Chloroform solvents mixture with a ratio of 1:9. The mixtures were stirred for 12 h at 150 rpm and 40 °C until full dissolution and formation of homogeneous solutions. The pure PSS and PBS dopes were prepared by mixing 7.5 wt% of the polymer. Lithium chloride (0.1 wt%) was added as an additive to the prepared membrane dopes. Each prepared dope was degassed for 1 h before being fed to the syringe and starting the electrospinning process.

#### Membrane preparation using electrospinning

The solution was drawn into a 10 mL syringe that was placed in a syringe pump for electrospinning. The electrospinning of the fibers was performed using (nano.01, MECC CO., LTD. JAPAN, Fukudo, Ogorishi, Fukuoka, Japan). The solution was first fed to the syringe with a stainless steel needle (12.3 mm inner diameter, OD: 0.9 mm, ID: 0.6 mm, MECC CO, Fukudo, Ogorishi, Fukuoka, Japan). The distance between the needle tip and the collector was kept at 25 cm, and an electric voltage of 20 kV was applied with a spinneret speed and widths of 50 mm/s and 120 mm, respectively. The flow rate of the polymeric solutions was 2.5–3.0 cc/h, and the electro-spinning time was 5 h^46^. The homemade collector was used; the collector was a flat carton sheet covered by aluminum foil. After completion of the electrospinning process, the membrane was immersed in distilled water at 40 °C for 2 h. Finally, the membrane was put in the oven at 60 °C for 1 day to remove the solvent residuals.

#### Adsorption/desorption of methylene blue dye

A stock solution of 10^–3^ M of Methylene Blue dye (MB) was prepared by dissolving an appropriate amount of the dye powder in 50 mL of distilled water. The required concentrations for carrying out various adsorption experiments were done by dilution. The effect of the prepared membranes on MB dye uptake % was examined. The following equation represented the amount of adsorption at equilibrium time q_e_ (mg/g).

1 $$ q_{e} = \frac{{(C_{0} - C_{e} )*V}}{W} $$

where C_0_ and Ce (mg/L) are the concentrations of dye at t = 0 min and at equilibrium time, respectively; V is the volume of dye solution (L); W is the mass of dry adsorbent used. The quantity of adsorption was measured at various time intervals from 0 to 180 min to investigate the kinetics of adsorption. Moreover, the thermodynamic parameters, the different modules of kinetics, and adsorption isotherms were applied. The values of adsorption amount at time t, qt (mg/g), and the dye removal (R %) were calculated by the following equations, respectively

2 $$ q_{t} = \frac{{(C_{0} - C_{t} )xV}}{W} $$

3 $$ \% R = \frac{{{\text{Co}} - {\text{Ct}}}}{{{\text{Co}}}}{ } \times {100} $$

Pseudo-first-order (PFO) and pseudo-second-order (PSO) kinetic models were most frequently employed to fit the collected experimental data and better understand the kinetics of the adsorption process. The linear forms of PFO and PSO can be described as been given in our previous works^40–45^.

#### Polymer and membrane characterization

The degree of polystyrene sulfonation is examined by determining the concentration of sulfur using a Varian atomic absorption spectrometer (AAS, Model: VARIAN AA240FS). Surface tension measurements were carried out using Krüss tensiometer (Pt ring method). Solution viscosity for blended polymer solutions was measured using Brookfield-programmable Rheometer-DV III URTRA. The chemical structures of the prepared samples were investigated by FTIR-ATR-VERTEX 80 (Germany). The instrument is equipped with a platinum Diamond disk with internal reflection in the range of 400–4000 cm^−1^, a resolution of 4 cm^−1^, and a refractive index of 2.4. The thermal stability of electrospinning membranes was studied using a Perkin Elmer thermogravimetric analyzer (TGA), with a heating range of 30 to 500 °C and a heating rate of 10 °C/min under N_2_ atmosphere. The adsorption of dyes on the best adsorbent was confirmed with JASCO V-730 Spectrophotometer with transmission mode of analysis in the range from 1000 to 200 cm^−1^ (λ_max_ for MB dye is 663 nm).

## Results and discussion

The synthesis of sulfonated polystyrene (SPS) was performed by a direct sulfonation reaction without a catalyst using concentrated sulfuric acid. This heterogeneous reaction was affected by many factors, such as acid concentration, temperature, and reaction time. The concentration of sulfur atoms is 20 mg/L, as determined by atomic absorption spectroscopy. The sulfonic group can be attached to the aromatic ring either in ortho- or para-positions. However, the substitution reaction often occurs in the para-position due to the steric effect (Fig. 1a,b).

The selection of the proper solvent for preparing polymer solutions suitable for electrospinning is a crucial step^47^. It was reported that in solutions of viscosity lower than 0.1 Pa, the morphology of the formed fibers was controlled by surface tension, and beads were more likely to be formed instead of fibers^48^. The preparation of SPS/PBS electrospun fibers was performed after examining the solvation of both polymers in suitable solvents. Although PBS has good solubility in chloroform (CHCl_3_), the low boiling point of CHCl_3_ and its rapid evaporation at the tip of the needle result in the solidification of the polymer inside the tip, which hinders the electrospinning process. On the other hand, polystyrene is soluble in both chloroform and 1,4-dioxane. After excessive testing of different co-solvent systems and varied sharing percentages, a co-solvent system of 9:1 (chloroform: 1,4-dioxane) was chosen for electrospinning the PBS/SPS polymer blend. Lithium chloride (LiCl) is added to the polymers’ solution to improve the electrical conductivity of the membrane dope, which is reflected in the solution polarity and improved electrospinning process. Table 1 shows the main parameters of the used solvents (chloroform: 1,4-dioxan)^49^.

**Table 1:** Characteristic parameters of chloroform and 1,4-Dioxan.

Solvent	Boiling point °C	Surface tension (mN/m)	Viscosity cP	Hansen solubility parameter (MPa1/2)
Chloroform	61.0	27.16	0.57	19.0
1,4-Dioxane	101.1	32.8	1.087	17.5

Many parameters affect the morphology of the electrospun fibers, among them the surface tension of the polymer solution. This property is strongly influenced by the polymer solution and solvent. The low surface tension of the spinning solution allows electrospinning to be carried out at a lower electric field^50,51^. Surface tension values of prepared polymer solutions shown in Table 2 are slightly higher compared to the values of pure solvents in Table 1. These results can be attributed to the low concentrations of the polymer blends used. Similarly, values of solution viscosity did not show a large change by doubling the ratio of PBS in the blended solution.

**Table 2:** Surface tension and viscosity values of blended solutions.

Sample	Surface tension (mN/m)	Viscosity (cP)
Solvent mixture	28.5	11
PSS/1% PBS	30	11.4
PSS/2% PBS	31	12

### Characterization of the fibers

#### FTIR-spectroscopy

**Figure 2:**
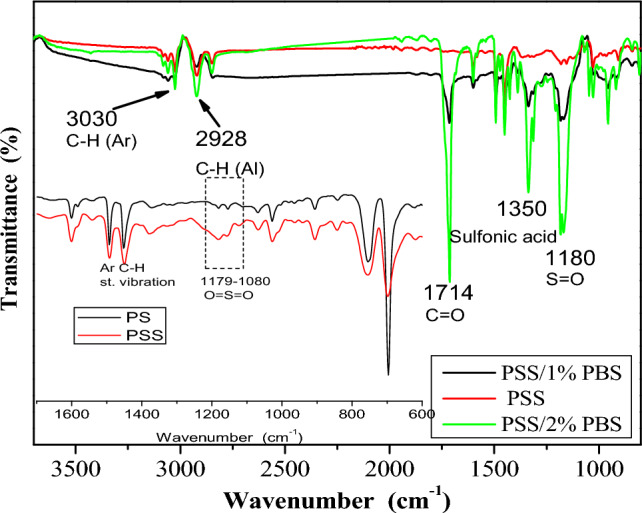
FTIR spectra of electrospun fibers.

The FTIR-ATR spectrum shown in Fig. 2 as an inset is further confirmation of the successful sulfonation of the PS. The band of aromatic C–H can be noticed at 3030 cm^−1^ while that for C–H aliphatic appears at around 2928 cm^−1^^52^. The band around 1450 cm^−1^ indicates the stretching vibration of aromatic C–H^53^. The bands around 1080–1170 cm^−1^ can be attributed to O=S=O overlapping with those for aromatic C–H deformation vibration of the PS^54^.

Investigation of the chemical structures of the fibers using ATR-FTIR was carried out, and the spectra are demonstrated in Fig. 2. The three samples showed two bands at 3030 and 2928 cm^−1^ corresponding to the stretching vibrations of C–H aromatic and aliphatic, respectively. (Fig. 2) Samples of PSS/1% PBS and PSS/2% PBS that contain PBS showed bands at 1714 cm^−1^ corresponding to C=O of the ester group^35^. The three samples depicted bands at 1350 cm^−1^ of the sulfonic group and 1180 cm^−1^ corresponding to the bending vibration of the C–O ester bond (Fig. 1c shows the chemical structure of PBS)^52^. As the intensity of these bands can be related to the amount of PBS in the blend, the intensity of C=O is more pronounced at 2% PBS sample. The same feature is noticed for the band of the sulfonic group (at 1350 cm^−1^).

#### Thermal analysis

**Figure 3:**
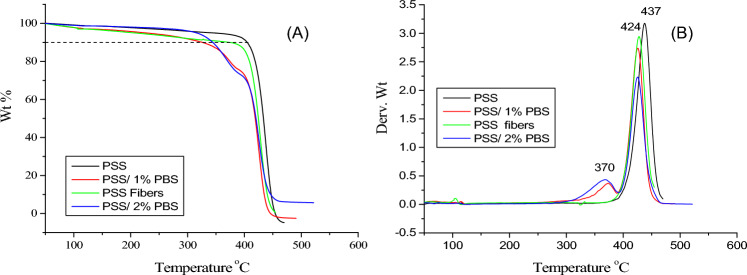
TGA thermogram of sulfonated polystyrene fibers and blends.

The thermal stability of the prepared fibers was examined using thermal gravimetric analysis. Figure 3A shows that PSS has the highest thermal stability compared with other samples (PSS fibers and fibers of PSS/PBS blends). Moreover, a 10% weight loss was observed at temperatures of 407, 383, 329, and 343 ºC for samples PSS, PSS fibers, 1% PBS, and 2% PBS, respectively. The lower thermal stability of the blends is due to the degradation of the aliphatic polyester. Nevertheless, no large difference in thermal stability between the blends could be observed since the increase in added PBS is small. The derivative curves shown in Fig. 3B confirmed the previous observation and demonstrated peaks at 370 ºC corresponding to the degradation temperature of both 1 and 2% PBS.

#### Investigation of film morphology

**Figure 4:**
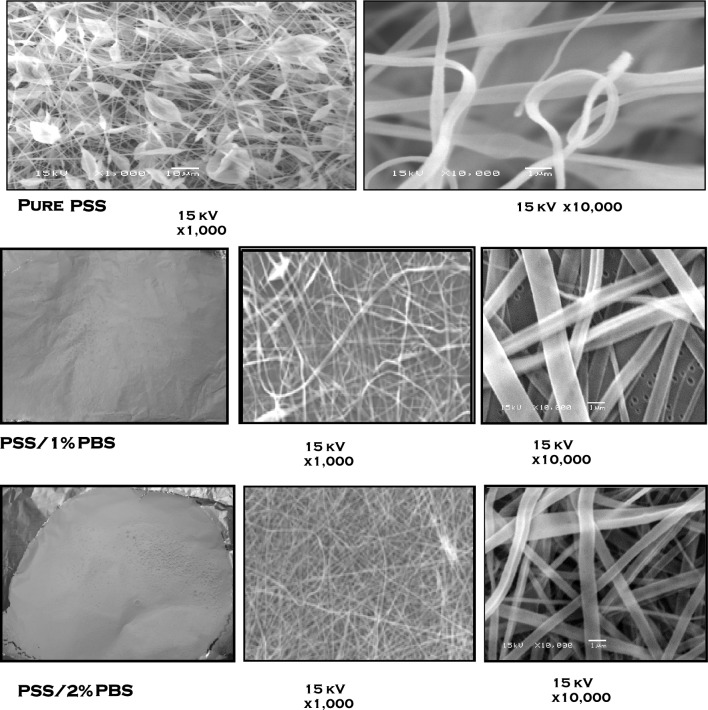
Photographs and SEM images of electrospun pure PSS and PSS/PBS blend in different concentrations.

Morphological investigation of pure PSS fibers showed that the formation of fibers connecting beads is prevalent, which can be attributed to the dryness of the fibers before reaching the collector, and the wet fibers tend to coalesce and form bundles. (Figure 4)^55^. Fibers of PSS carry ionic groups arranged along the polymer chains; hence, repulsive forces arise, and the polyelectrolyte can have an extended conformation. The addition of PBS to the polymer solution decreased the repulsion and changed the conformation of the chain, causing chain entanglement. Other reports illustrated that chain entanglements are required to counter capillary instability before fiber formation during electrospinning^13,56^. By observing the SEM images depicted in Fig. 4, it is obvious that fiber quality is improved significantly by increasing the amount of PBS in the blend solution. Moreover, continuous and more homogenous fibers are produced using the sample PSS/2% PBS. This improved fiber quality can be attributed to the increased viscosity of the blended solution. Subramanian et al*.* reported that lower solution viscosity is responsible for the formation of beads and fine fibers, while smoother and thicker fibers can be formed at higher viscosities^15^. Moreover, higher concentrations increase solution viscosity, which enhances fiber entanglements and stabilizes the fibers^57^.

### Dye removal investigations

For the degradation of MB solution under UV light, the photocatalytic activity of all samples was considered. First, a photolysis control investigation was conducted for 120 min. under UV light without the prepared samples. The MB concentration did not change over time, indicating that MB is a non-biodegradable dye.

#### Influence of pH

**Figure 5:**
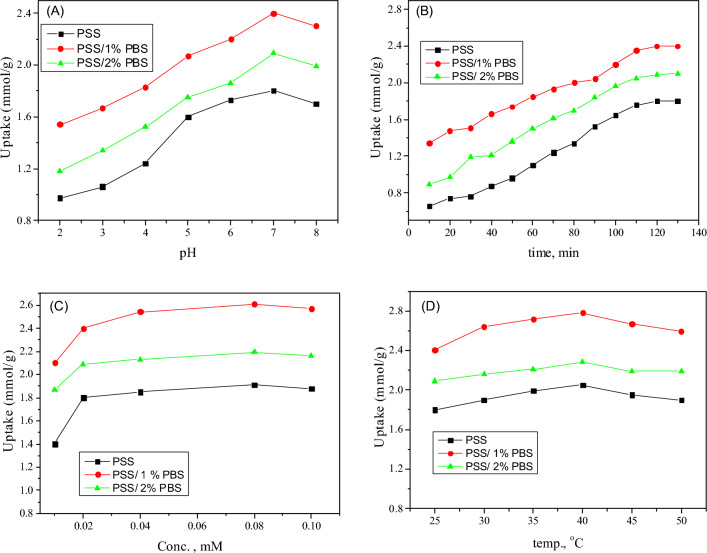
Effects of (**A**) pH, (**B**) contact time, (**C**) adsorbents concentration, and (**D**) temperature on the uptake of MB by PSS, PSS/1% PBS, and PSS/2% PBS.

The surface charges of solids and subsequently the surface binding sites are obviously influenced by the pH of the solution, and affecting the adsorption efficiency^43–45^. The data obtained are given in Fig. 5A. Inspection of Fig. 5A revealed that adsorption capacity is increased by raising the solution pH up to 7, while at pH values higher than 7 the adsorption capacity is diminished. The obtained results can be attributed to the assumption that the acidic medium (at pH lower than 7) increases the active sites of the solids, and consequently, the negative centers (SO_3_^−^) of the dye are increased. This might be responsible for facilitating the adsorption process. However, the decrease in adsorption in alkaline medium (at pH values higher than 7) is due to the repulsion force between the ionized dye molecule and the adsorbed OH^−^, which agrees with the results reported by Mittal et al.^58^ The found data revealed that the presence of PBS (1%) in the nanofibers’ blend increased the adsorption of MB. The main reason for the adsorption might be the driving force. The mechanism of adsorption might be the formation of an n–π interaction between the C=O group (as an electron donor group carrying pairs of electrons on the oxygen atom) and the phenyl rings of MB (as acceptors)^59,60^. The increase in the percentage of PBS (2%) resulted in lower adsorption uptake. This behavior might be attributed to the increase of the sulfonic groups that are repulsive to the same group present in the dye molecule (c.f. Figure 1). Another suggestion for the adsorption mechanism might be due to the π−π electron donor–acceptor interactions of aromatic compounds. So, at a higher pH value (> 7) there was a full overload of all (–OH) functional groups, which might be the reason for the decrease in adsorption. The lower adsorption in the case of the sample containing 2% PBS can be due to the presence of a larger amount of PBS, which might hinder the penetration of the dye in order to attach with PPS as adsorption occurred through a multilayer process as will be discussed in Sect. (3.3.5). Also, as has been discussed, the utilization of higher amounts of PBS increased the viscosity and consequently increased the accumulation of particles, which decreased light transmission to the catalyst's surface and thus reduced the quantity of reaction-accessible active surface.

#### Influence of contact time

The influence of contact time on the adsorption of MB was investigated at natural pH and room temperature, as shown in Fig. 5B, and the data was collected over a period of 10–130 min. The results revealed that increasing the contact time led to enhanced adsorption of MB, and the equilibrium was reached after 120 min. It can be suggested that during the initial step vacant surface sites for adsorption existed. However, over time, the vacant sites for adsorption decrease and repulsive forces between dye molecules and adsorbate MB on the surface of solids arise. These results reflect the relationship between the number of free sites and the adsorption rate, as discussed in our previous work^60^. The long time (120 min) might result in steric hindrance to the attraction between the adsorbent and dye molecules.

#### Influence of concentration

The uptake in mmol/g of various concentrations (10–100 ppm) of MB over different solids is given in Fig. 5C. The obtained data revealed that uptake is highly increased in the concentration of MB between 10 and 20 ppm. Contrary to the trend, almost no notable change was found at high concentrations (30–100 ppm). This behavior can be attributed to the increasing dye concentration accompanied by the change in solution viscosity. A layer is formed on the outside surface, which blocks the pores and/or decreases the diffusion of solution through the adsorbent^59,60^.

#### Influence of temperature

Figure 5D shows the uptake of MB by different prepared solids at temperatures ranging from 25 to 50 °C. It is obvious from Fig. 5D that increasing temperature from 25 to 40 °C led to an increase in dye uptake. This behavior might be attributed to the diffusion of dye molecules from the bulk to the surface of the solid adsorbent. The opposite trend observed at high temperatures > 40 °C is due to the release of dye adsorbed on the solid surface into the aqueous solution, which destroys certain active sites. Consequently, it led to bond breaking on the adsorbent surface and/or a decrease in sorption power among the dye molecules and energetic sites of the prepared solids^43,44^. The obtained results showed that low temperatures were favorable for dye sorption over prepared solids.

#### Adsorption isotherms

**Figure 6:**
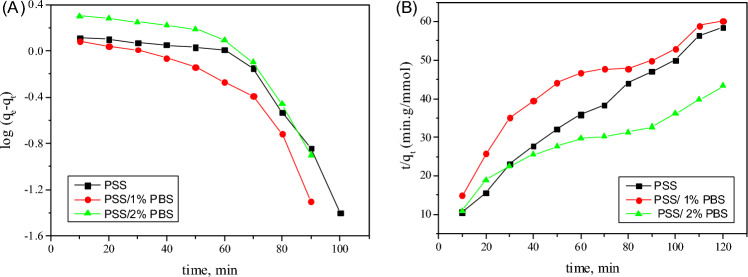
A: Pseudo-first order (PFORE) B: Pseudo second order (PSORE).

The Langmuir and Freundlich isotherm models are used to study the adsorption of MB dye over different as-prepared adsorbents (Table 3). Inspection of the data reveals that the adsorption of MB over various as-prepared solids fits with the Freundlich isotherm as R^2^ is close to one. This result indicates that the adsorption of the dye molecules has occurred through multilayer coverage on the surface of the adsorbent. K_f_ values are in the order PSS/1% PBS > PSS > PSS/2% PBS, which indicates the superiority of adsorptive power. (Fig. 6) This order could be related to the presence of new functional groups (C=O) that act as active sites for adsorption and the ease of penetration of the dye through a multilayer of adsorbent. However, the increase in PBS (PSS/2% PBS) concentration prevented the dye penetration.

**Table 3:** Isotherm model constants and correlation coefficient for MB adsorption by PSS, PSS/1% PBS and PSS/2% PBS.

Isotherm model	Parameters	PSS	PSS/1% PBS	PSS/2% PBS
Langmuir	q_max_ (mmol/g)	4.6	6.4	3.8
	K_L_ (L/g)	86.2	120.4	81.5
	R^2^	0.973	0.984	0.956
	χ^2^	0.015	0.014	0.013
Freundlich	K_f_ (mmol/g)	3.1	4.2	1.9
	1/n	0.5	0.4	0.9
	R^2^	0.995	0.998	0.993

#### Adsorption kinetics

Parameters of adsorption kinetics for various models of MB over different as-prepared solids are given in Table 4. The obtained data depicts that adsorption of MB has occurred according to pseudo-second order. This is confirmed as the q_e_ calculated from pseudo-second-order is almost equal to the experimental data and also R^2^ (correlation coefficient) is > 0.94 higher than that calculated for the pseudo-first-order model. The obtained data confirmed that the chemisorption step is the rate-determining step^61^.

**Table 4:** Kinetic model constants and correlation coefficient for MB adsorption by PSS, PSS/1% PBS, and PSS/2% PBS.

Kinetic model	Parameters	PSS	PSS/1% PBS	PSS/2% PBS
Pseudo-first order model	q_e_^exp^ (mmol/g)	4.2	5.1	3.8
	q_e_ (mmol/g)	1.9	1.5	1.3
	K_1_ (min^−1^)	0.017	0.04	0.009
	R^2^	0.88	0.85	0.78
Pseudo-second order model	K_2_ (g/mmol·min)	4.4	5.8	3.2
	q_e2_ (mmol/g)	0.017	0.016	0.017
	R^2^	0.984	0.991	0.979

 The capacity of the photocatalyst to bind dye molecules and the degree to which the recombination of (e^−^ + h +) pairs is influenced determine the photocatalytic performance most frequently. Here, the presence of PBS in samples can increase their specific surface area, increase the efficiency with which light is absorbed by their surface, and have the potential to hasten the transfer of (e^−^ + h +) pairs of PSS, improving the photocatalytic activity^60^. Based on the earlier results, the mechanism of the photocatalytic degradation of MB dye on the prepared samples is illustrated in Eqs. (4–12).

The first step is to create the (e^−^ + h +) pairs on the prepared samples under the action of UV-light, which will excite the electrons into the conduction band (CB) while keeping the holes in the valence band (VB), as described in Eq. (4). The photogenerated e^−^ on the CB of PSS/PBS may migrate to the CB of PSS rather than returning to the VB, delaying the recombination of the photogenerated pairs (as illustrated in Eqs. (5–7)). Otherwise, it may combine with the dissolved oxygen to form superoxide radicals^61^. According to Eqs. (8 and 9), the produced superoxide radicals will change into hydroxide radicals by reaction with water. Additionally, as the adsorbed water molecules react with the hydroxyl radicals at the PSS VB, more hydroxyl radicals are produced Eq. (9). According to Eqs. (11 and 12) the produced hydroxyl and superoxide radicals are strong oxidizing species and photodegrade the MB into CO2, water, and non-toxic compounds.

4 $$ {\text{PSS}}/{\text{PBS}} + {\text{h}}\upsilon \to {\text{PSS}}/{\text{PBS}}\,\left( {{\text{h}} + {\text{VB}} + {\text{e}}^{ - } {\text{CB}}} \right) $$

5 $$ {\text{O}}_{{2}} + {\text{PSS}}/{\text{PBS}}\, \left( {\text{e CB}} \right)\, \to \,{\text{O}}_{{2}} \cdot $$

6 $$ {\text{PSS}}\,\left( {{\text{e}}^{ - } {\text{ CB}}} \right) \to {\text{PBS}}\,\left( {{\text{e}}^{ - } {\text{ trap}}} \right) $$

7 $$ {\text{O}}_{{2}} + {\text{PBS}}\,\left( {{\text{e}}^{ - } {\text{ trap}}} \right) \to {\text{O}}_{{2}} \cdot $$

8 $$ {\text{O}}_{{2}} \cdot + {\text{2HO}} \cdot + {\text{H}}^{+} \to {\text{H}}_{{2}} {\text{O}}_{{2}} + {\text{O}}_{{2}} $$

9 $$ {\text{H}}_{{2}} {\text{O}}_{{2}} \to {\text{2OH}} \cdot $$

10 $$ {\text{H}}_{{2}} {\text{O ads}}. + {\text{PSS}}\,\left( {{\text{h}} + {\text{VB}}} \right) \to \cdot {\text{OH}} $$

11 $$ {\text{O}}_{{2}} \cdot + {\text{MB ads}}. \to {\text{CO}}_{{2}} + {\text{H}}_{{2}} + {\text{non}} - {\text{toxic products}} $$

12 $$ {\text{OH}} \cdot + {\text{MB ads}}. \to {\text{CO}}_{{2}} + {\text{H}}_{{2}} {\text{O}} + {\text{non}} - {\text{toxic products}} $$

#### Activation Energy (E_a_)

The suggestion of the adsorption process-whether a physical or chemical process-can be decided from the value of E_a_^62,63^. The values of activation energy for MB adsorption were found to be 36.98, 30.70, and 43.40 kJ/mol over PSS, PSS/1% PBS, and PSS/2% PBS, respectively. The obtained data emphasizes that adsorption has occurred physically, which agrees with the data acquired by the Langmuir and Freundlich isotherm models.

#### Adsorption thermodynamics

The effect of temperature on the adsorption of MB could be evident by studying adsorption thermodynamic parameters such as standard enthalpy change (ΔH°). This parameter was calculated from Gibbs free energy equations for the adsorption of different samples at 25, 35, and 40 °C^64,65^. The calculated values of ΔH° for MB adsorption on PSS/1% PBS, PSS/2% PBS, and PSS were 65.65, 57.54, and 23.80 kJ/mol, respectively. The positive value of ΔH° reflects that the adsorption process is endothermic, which is confirmed by increasing adsorption by elevating the temperature to 40 °C.

#### Comparison with other adsorbents

A comparison of the highest adsorption uptake of MB over various as-prepared solids with other adsorbents described in the literature is given in Table 5 to show the efficiency of as-prepared solids in dye removal^8,40,59,66–68^. The data given in Table 5 reveal that the as-prepared solids have a higher adsorption capacity for MB compared with other adsorbents. These results draw attention to the as-prepared solids as promising materials for dye removal from an aqueous solution. The comparison was carried out with the sample treated with PSS/1% PBS, as it has the highest uptake. The highest adsorption capacity of PSS/1% PBS might be due to the existence of greater surface imperfections, which greatly improve charge separation and boost light absorption capacity. The PSS/1% PBS sample has a lower recombination rate than the other samples.

**Table 5:** Comparison of MB dye removal efficiency using PSS, PSS/1% PBS and PSS/2% PBS via adsorption.

No	Adsorbent	Max. adsorption capacity, mol	Ref
1	CMC/ZSM-5/ZIF-8	10.49	^66^
2	MCMFCs	303	^67^
3	PSS/SiO_2_	84	^8^
4	PSS/Fe_3_O_4_	46.6	^40^
5	**C**M	9.1	^59^
6	CMC-coated Fe_3_O_4_@SiO_2_ MNPs	29	^68^
7	PSS/1% PBS	846	Present work

#### Reuse of the adsorbents

**Figure 7:**
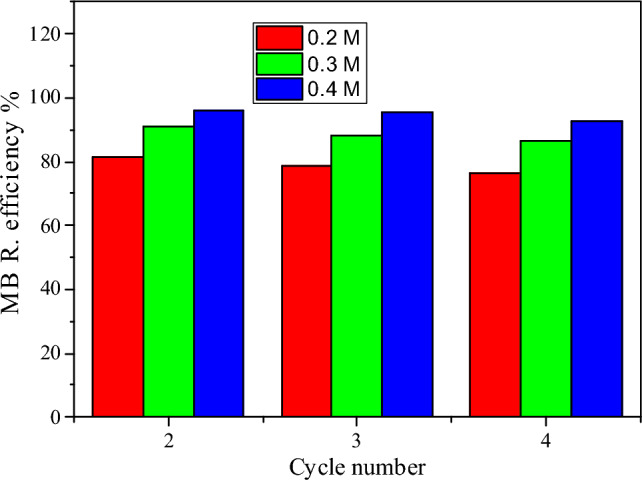
Regeneration of PSS/1% PBS toward adsorption of MB dye using various concentrations of nitric acid.

As sample PSS/1% PBS provided the highest uptake, an investigation of its reuse ability is carried out. Figure 7 shows four times the reuse of PSS/1% PBS for MB uptake from an aqueous solution, later being treated with sodium hydroxide solution as a desorbing agent. It was found that PSS/1% PBS is capable of dye removal even after usage several times. The deactivation of the prepared sample is most likely attributed to the surface active sites becoming inactive when aggregate particles are formed. As a result of subsequent exposure to dye molecules during recycling processes, the intermediate compounds generated as a result of the photodegradation process are increased, consuming some active radicals necessary for interacting with the dye molecules.

## Conclusion

The main target of this work is the utilization of polystyrene waste for the preparation of electrospun fibers from blends of polystyrene sulfonate (PSS) and polybutylene succinate (PBS) as a promising membrane for the removal of MB dye. Sulfonation of polystyrene is successfully carried out and confirmed by atomic absorption spectroscopy and FTIR. Blends of PSS and PBS containing 1 or 2% of the latter polymer were prepared by dissolving them in a chloroform/1,4-dioxane mixture. The morphology study of the formed films showed that the blend with a higher PS ratio possessed bead-free fibers with homogenous diameter, which can be attributed to the increased hydrophilicity of the polymer blend. Electrospun membranes were used as adsorbents for MB dye under the influence of variable conditions such as pH, contact time, dye concentration, and temperature. The obtained data revealed that PSS/1% PBS has the highest uptake and good regeneration efficiency. Investigation of various kinetic models demonstrated that the adsorption is an endothermic pseudo-second-order process that fits the Freundlich isotherm model. All collected results foresee that PSS/1% PSB is a promising material for dye removal from an aqueous solution. Moreover, the prepared membrane is ecofriendly since it is based on a waste material and a biodegradable polymer.

## Data Availability

All data generated or analyzed during this study are included in this article.
